# Association of Childhood Socioeconomic Status with Leukocyte Telomere Length Among African Americans and the Mediating Role of Behavioral and Psychosocial Factors: Results from the GENE-FORECAST Study

**DOI:** 10.1007/s40615-021-01040-5

**Published:** 2021-05-04

**Authors:** Rumana J Khan, Belinda L Needham, Shailesh Advani, Kristen Brown, Casey Dagnall, Ruihua Xu, Gary H. Gibbons, Sharon K. Davis

**Affiliations:** 1 Cardiovascular Section, Social and Behavioral Research Branch, National Human Genome Research Institute, National Institutes of Health, 10 Center Drive, Room 7N316 MSC 1644, Bethesda, MD USA; 2 Department of Epidemiology, School of Public Health, University of Michigan, 1415, Ann Arbor, Washington Heights, MI USA; 3 Cancer Genomics Research Laboratory, Division of Cancer Epidemiology and Genetics, National Cancer Institute, Bethesda, MD USA; 4 Cardiovascular Disease Section, Metabolic, Cardiovascular and Inflammatory Disease, Genomics Branch, National Human Genome Research Institute, National Institutes of Health, Bethesda, MD USA; 5 National Heart, Lung, and Blood Institute, National Institutes of Health, Bethesda, MD USA

**Keywords:** Childhood socioeconomic status, Telomere length, African American, Mother’s education, Mediator, Path analysis

## Abstract

**Purpose:**

We examined if childhood socioeconomic status (SES) was related to adult leucocyte telomere length (TL) using the data of 361 African American (AA) participants from the GENE-FORECAST Study. We also assessed the mediating role of behavioral and psychosocial factors in the association between childhood SES and adult TL.

**Methods:**

Childhood SES was assessed individually by using participant’s mother’s education and occupation, father’s education and occupation, parental home ownership, and family structure. TL was assessed using the quantitative polymerase chain reaction method. Information on potential confounders and mediators were collected. The associations of childhood SES with TL were assessed using multivariable linear regression models. We used path analysis to quantify and test the share of these associations that was statistically explained by each of the mediators (participant’s educational attainment, smoking status, physical activity, dietary habit, perceived stress, and depressive symptoms).

**Results:**

Mother’s education was associated with longer average TL (β: 0.021; 95% CI: 0.001, 0.04, *p*=0.038) in confounder adjusted models. Once mediators were introduced in the model, the estimates were reduced and remained marginally significant (β: 0.017; 95% CI: −0.003, 0.038, *p*=0.061). According to path model, approximately 19% of the effect of mother’s education on TL (β: 0.004; 95% CI: −0.001, 0.01, *p* < 0.10) was mediated through participant’s own education level. No significant mediation effect was observed for any other mediators.

**Conclusions:**

These data provide evidence that participant’s mother’s education was positively linked to adult TL in AA population. Participant’s own educational level partially explained this association.

**Supplementary Information:**

The online version contains supplementary material available at 10.1007/s40615-021-01040-5.

## Introduction

Age-related chronic diseases are disproportionately prevalent among socioeconomically disadvantaged individuals [1]. Compared to their White counterparts, African American (AA) population are generally at higher risk for almost all age-related chronic diseases according to the Office of Minority Health, Department for Health and Human Services [2]. Although health outcome research mostly is focused on adult socioeconomic status (SES), accumulating data implicates that disadvantageous SES during childhood also contributes to greater risk of age-related chronic diseases [3, 4]. Pathogenesis of chronic diseases is directly associated with cellular aging. Telomeres, which are nucleoprotein structures at the ends of chromosomes, play a critical part in cellular aging and may contribute to development of chronic disease [5, 6]. Telomeres stabilize chromosomes and maintain genome and cellular integrity [7]. Telomeres generally shorten progressively with every cell division, and thus, telomere length typically declines with advancement of age [8]. Telomeres can also shorten due to damage caused by oxidative stress and inflammation [9, 10]. Oxidative stress and inflammation can be triggered by a wide range of environmental, lifestyle or health behavior, psychosocial, and social factors [9, 11]. As a result, the influence of lifestyle factors like lack of physical activity, cigarette smoking, poor sleep, and poor nutrition on telomere shortening have been largely studied [12–17]. Shorter telomeres have also been linked to psychosocial stress, and depression disorders in large-scale epidemiologic studies [18–21]. A recent meta-analysis of thirty-eight studies reported telomere length to be significantly negatively correlated with lifetime depression [22]. The relationship between greater perceived stress and shorter telomere length was also reported in several studies [23–25]. While most studies have noted a pattern of shortening of telomere with depression and stress, there are also reports that do not support this finding [26, 27]. Since SES persistently impacts all these lifestyle and psychosocial factors, several studies have assessed the link between adult SES and telomere length [28–33]. The evidence for an association has been mixed. Some studies have shown lower SES to be associated with shorter telomere length [29–31] whereas others have shown little or nonsignificant association [28, 32, 33]. Since childhood SES is predictive of adult SES [34], more recently, a few studies examined the direct association between disadvantageous social factors during childhood and shortening of telomere to see if telomere is one of the links between early life SES and adulthood disease [31–33, 35–37].

Different interrelated conceptual models have been proposed to explain the longstanding effect of childhood SES on subsequent age-related chronic disease morbidity and mortality: timing, pathway, accumulation, and change model [38–40]. The timing model posits that childhood is a sensitive developmental period when individuals are most vulnerable to the negative health-related consequences of low SES. According to this model, childhood SES is associated with increased chronic disease morbidity and mortality, independent of adult SES. Alternatively, in the pathway model, early life events and environments influence later life experiences, opportunities, and health risk factors. This life course model suggests that childhood SES affects adult health through its association with adult SES, and other risk factors, such as increased stress, physical inactivity, smoking, and poor diet. Next, the accumulation model suggests that the health-damaging effects of low SES accumulate over time with increasing intensity and duration of exposure. Smith et al. suggest that if factors operating at different life stages are combined, large differences in cardiovascular disease risk will be observed [33]. Finally, the change or the social-mobility model represents the fact that an individual’s SES is dynamic, and it incorporates the trajectory of socioeconomic mobility across one’s lifetime in determining disease risk. For example, downward or low SES trajectories from childhood to adulthood could be associated with increased disease risk compared with an upward or high SES trajectory

Studies linking childhood SES and telomere length have mostly focused on single indicators of SES, such as either parental education or home ownership or perceived social class [31–33, 35–37]. Therefore, the association between comprehensive SES measures and adult telomere length often remains unknown. Given the multidimensional nature of SES, addressing childhood SES from a multiple perspective can provide new insight. Furthermore, despite having higher prevalence of age-related chronic disease, and the overall lower socioeconomic position compared to Whites, AA participants were included only in few of these studies [31, 36], yet none reported the association separately for them. More research exclusively in AA population is especially needed given recent studies indicating a faster decline in telomere length with age in African Americans than in Whites [41–43]. Cumulative exposure to multiple psychosocial stressors over the life course has been suggested as possible contributors to this faster shortening [41]. Therefore, understanding the relationships between childhood SES and telomere length has the potential to elucidate the cellular mechanisms by which early life stressors contribute to life-shortening diseases in AA population.

In this study, we examined if childhood SES as measured by parental education and occupation, house ownership, and family structure were individually related to adult telomere length (TL) using the data of 361 AA participants from the GENE-FORECAST (Genomics, Environmental Factors, and the Social Determinants of Cardiovascular Disease in AA) Study. The current analysis tested pathway model and explored potential mediating pathways linking childhood SES and telomere shortening including adult health behavior (smoking, dietary habit, and physical inactivity), psychological factors (depressive symptoms and perceived stress), and participant’s own educational attainment. As a secondary analysis, the study also investigated the impact of social mobility on adult TL.

## Methods

### Data Source

The GENE-FORECAST study is a community-based cross-sectional study initiated in 2014 to investigate the genetic, social, and environmental determinants of cardiovascular disease risk factors and phenotypes in self-identified, US born AA men and women. This is an ongoing study, which to date has recruited over 550 participants aged 21–65 years, residing in the metropolitan Washington DC, Montgomery County, and Prince George’s County area. There were 392 participants for whom both telomere and childhood SES data were available. In the current analyses, 31 participants were excluded because they did not have information on other required covariates (Supplement Table 2). The GENE-FORECAST study was approved by the Institutional Review Board of the National Institutes of Health Office for Human Subjects Research Protections (14-HG-0048); and the participants provided written informed consent.

### Assessment of Childhood SES

Six aspects of childhood SES were derived from an interviewer-administered questionnaire (Supplementary Table 1). Participants were asked to think back on the time while they were growing up until age 16 years and information was obtained on father’s educational attainment, father’s occupation, mother’s educational attainment, mother’s occupation, parental home ownership, and family structure. Each aspect of childhood SES was used as a continuous variable where higher values corresponded to higher SES. Details of the questions and scoring methods for assessment of childhood SES are summarized in Supplementary Table 1. In brief, scores were derived for father’s and mother’s education (range 0 (least, 8th grade or below) to 7 (most, Doctorate degree)), father’s and mother’s occupation (range 0 (nonprofessional work) to 1 (professional or managerial work)), parental housing (range 0 (least, lived with relatives/others) to 2 (most, had own house)), and family structure (range 0 (least, raised by someone other than parents) to 3 (most, were raised by both parents)). The individual SES measures were also examined as categorical variable as sensitivity analysis (Supplementary Table 1).

### Telomere Length Measurement

Relative telomere length measurement was performed on DNA isolated from peripheral blood leukocytes at the Cancer Genomics Research laboratory, National Cancer Institute, using an assay adapted from Cawthon’s published protocol [44, 45]. TL was determination by quantitative polymerase chain reaction (qPCR) measures and ratio of telomere (T) signals, to autosomal single copy gene (36B4 S) signals (T/S), was calculated using the LightCycler software (Release 1.5.0, Roche, Indianapolis, IN, USA). This raw T/S ratio was normalized by internal control DNA samples to yield relative standardized T/S ratios. The detailed laboratory and quality control procedure is summarized in supplementary section. In this study, the intra-class correlation coefficient (ICC) between the repeated measures was 0.902 (95% confidence interval 0.885, 0.928) and the mean coefficient of variation (CV%) for the technical replicates standardized T/S measures was 4.90%.

### Measurement of Covariates

Details on covariates and their assessment procedure are listed in Supplementary Table 2. Demographic factors, including age (years), gender, and marital status (married/partnered vs. single/divorced/widowed), were self-reported by participants. Body mass index or BMI (weight in kg/height in meter^2^) was calculated. BMI 25.0 to <30 was defined as overweight and BMI 30.0 or higher as obesity. Information on existing chronic disease conditions (yes/no) including diabetes and hypertension were obtained.

Proposed mediators included participant’s own educational attainment, smoking status, physical activity, dietary habit, perceived stress, and depressive symptoms. Measurement procedures are detailed in Supplementary Table 2. Participant’s educational attainment, smoking behavior, physical activity [38], and dietary data [46] were self-reported. The 20-item Center for Epidemiologic Studies Depression Scale (CES-D) was used to assess the presence of depressive symptoms [47]. Perceived stress was assessed using the 10-item self-reported Perceived Stress Scale (PSS) questionnaire [48]. The internal consistency of the PSS and Depression instruments were analyzed with Cronbach’s alpha, which yielded coefficient of 0.863 and 0.772, respectively.

## Statistical Methods

Descriptive statistics were used for summarizing characteristics of the participants. Age-adjusted regression coefficients for demographic characteristics, childhood SES indicators, health behaviors, and health status indicators with TL were first examined. Next, we examined the associations of each childhood SES measures (father’s educational attainment and occupation, mother’s educational attainment and occupation, parental home ownership, and family structure) with TL using multivariable linear regression models.

We fitted 2 models for each regression model. Model 1 was adjusted for potential confounders (participant’s age, sex, marital status, obesity, and existing health conditions). Model 2 additionally was adjusted for prespecified intervening or mediator variables including health behavior (smoking, dietary habit, and physical inactivity), psychological factors (depressive symptoms and perceived stress), and participant’s own educational attainment. Model 1 and 2 were run separately for each childhood SES indicator.

Supplementary Figure 1 shows the proposed path analysis model through which childhood SES may impact adult telomere length. Briefly, our model postulated direct and indirect paths from childhood SES to telomere length through several intervening variables (smoking, dietary habit, and physical inactivity, depressive symptoms, perceived stress, and participant’s own education). As a sensitivity analysis, we also tested Supplementary Figure 2 to examine if the outcome changed by adding plausible pathways between participant’s own education and their lifestyle factors (smoking, dietary habit, and physical inactivity, depressive symptoms, perceived stress). To investigate the extent to which the intervening variables mediate the association between childhood SES measures and telomere length, we performed path analysis by using analysis of moment structure (AMOS). Detail analysis procedure is summarized in the supplementary section.

The social-mobility framework was tested using life-course education trajectories. The analyses used parent’s educational level as a measure of childhood SES and compared trajectories with participant’s educational level. Each education measure was dichotomized as follows: parent’s educational level, low (high school graduate or less) vs. high (some college or above); participant’s educational level, low (some college or less) vs. high (4 year college or higher); These categorizations were based on previously used theoretical foundations of trajectories in life-course epidemiology [39]. Four potential SES trajectories were investigated: stable high SES (high childhood and high adulthood), declining SES (high childhood and low adulthood), increasing SEP (low childhood and high adulthood), and stable low SES (low childhood and low adulthood).

## Results

### Sample Characteristics

Our final study sample comprised 361 participants that had complete data for all variables. Participant’s demographic characteristics, childhood SES indicators, health behaviors, and health status indicators distributions with age-adjusted regression coefficients for associations with TL are detailed in Tables 1 and 2. Mean age of our participants was 48.50 years (SD = 11.46; range =22 to 65). The majority of our participants were females (70.6%, *n*= 255), 30.5% were married or partnered (*n* =110), and 62.9% reported to have four-year college education or beyond. In this sample, almost 87% were either overweight or obese, while about 9% and 41.6% reported having diabetes and hypertension, respectively. In terms of childhood SES indicators, mothers of participants were slightly more likely to have higher education than fathers of the participants (i.e., 29% of mothers having 4 year college or higher education vs. 24.4% of fathers). More than two-thirds of the participant’s parents owned a house. About 22% were raised by a single mother compared to only 4.2% by a single father. Most of the participants, however, were raised by both parents. Age was significantly inversely associated with TL (*p* < 0.001). In age-adjusted models, being female, participant’s higher education, and their mother’s higher education were associated with longer TL (*p* < 0.05).

**Table 1:** Descriptive statistics of demographic characteristics and childhood SES indicators of study participants with age-adjusted mean differences (95% CI) in TL (*n*=361): the GENE-FORECAST study

	Percentage/mean (SD)	Frequency	^1^Age adjusted mean difference in TL, β (95% CI) *p*
Age	48.50 (11.46)		
Gender female	70.6	255	0.105 (0.026 to 0.184) 0.009
Married/partnered	30.5	110	−0.01 (−0.09 to 0.07), 0.875
Participant’s education			
0–12 years/high school diploma	9.7	35	Reference
Some college	27.4	99	0.08 (−0.053 to 0.214) 0.234
4 year college or higher	62.9	227	0.16 (0.036 to 0.283) 0.012
Childhood socioeconomic status			
Father’s education			
0–12/ high school diploma	61.8	223	Reference
Some college	13.9	50	0.047 (−0.06 to 0.156) 0.387
4 year college or higher	24.4	88	0.021 (−0.067 to 0.11) 0.638
Mother’s education			
0–12/high school diploma	54.3	196	Reference
Some college	16.6	60	0.135 (0 .033 to 0.238) 0.01
4 year college or higher	29.1	105	0.076 (−0.01 to 0.162) 0.071
Father’s occupation			
Professional/managerial	36.6	132	−0.005 (−0.081 to 0.071) 0.884
Mother’s occupation			
Professional/managerial	35.2	127	−0.014 (−0.091 to 0.062) 0.709
Home ownership			
Lived with relatives/other	1.4	5	Reference
Lived in a rented house	29.6	107	0.192 (−0.12 to 0.51) 0.23
Lived in own house	69.0	249	0.195 (−0.12 to 0.51) 0.21
Family structure/raised by			
Someone other than parents	3.2	12	Reference
Single father	4.2	15	0.239 (−0.032 to 0.51) 0.098
Single mother	22.2	80	0.098 (−0.122 to 0.317) 0.38
Both parents	70.4	254	0.149 (−0.061 to 0.36) 0.16

*SES* socioeconomic status, *TL* leukocyte telomere length

^1^Values are age-adjusted regression (β) coefficients (95% CIs) and *p* values for associations of demographic characteristics and childhood SES with TL

**Table 2:** Health behavior and disease conditions of study participants with age-adjusted mean differences (95% CI) in TL by health behavior and disease conditions (*n*=361): the GENE-FORECAST study

	Percentage/mean (SD)	Frequency	Age adjusted mean difference in TL, β (95% CI) *p*
Smoking status			
Never	78.1	282	Reference
Past	15.0	54	−0.003 (−0.11 to 0.09) 0.943
Current	6.9	25	−0.029 (−0.17 to 0.12) 0.69
Physical activity during leisure			
Much less than others	6.4	23	Reference
Less than others	36.0	130	0.064 (−0.09 to 0.22) 0.417
The same as others	41.6	150	0.095 (−0.06 to 0.24) 0.226
More than others	11.1	40	0.143 (−0.04 to 0.32) 0.118
Much more than others	5.0	18	0.031 (−0.19 to 0.24) 0.78
Dietary habit			
Poor	8.3	30	Reference
Fair	36.8	133	−0.034 (−0.17 to 0.11) 0.645
Good	36.8	133	−0.024 (−0.17 to 0.12) 0.733
Very good	15.2	55	0.066(−0.09 to 0.22) 0.417
Excellent	2.8	10	0.015 (−0.27 to 0.24) 0.531
Hypertension	41.6	150	−0.06 (−0.14 to 0.02) 0.12
Diabetes	8.9	32	0.014 (−0.11 to 0.14) 0.829
^2^Obesity/overweight			
Normal	13.30	48	
Overweight	32.96	119	0.047 (−0.071 to 0.16) 0.435
Obesity	53.74	194	−0.01 (−0.112 to 0.109) 0.872
^3^Depressive score	8.41 (7.2)		0.003 (−0.93 to 6.62), 0.16
^4^PSS	12.57 (6.41)		0.004 (−0.001 to 0.01), 0.12

*TL* telomere length, *PSS* Perceived Stress Score

^1^Values are age-adjusted regression (β) coefficients (95% CIs) and *p* values for associations of health behavior and disease conditions with TL

^2^Overweight was defined as body mass index (BMI) greater than or equal to 25 and obesity as BMI greater than or equal to 30

^3^Depressive score was calculated from the 20-item Center for Epidemiologic Studies Depression Scale (CES-D)

^4^PSS was assessed using 10-item self-reported Perceived Stress Scale (PSS) questionnaire

### Childhood SES, TL, and mediation

Table 3 shows adjusted mean differences of TL associated with childhood SES measures. Estimates correspond to mean differences in T/S ratio of TL per each unit increase in childhood SES component. After controlling for age, gender, marital status, and health conditions, such as BMI, diabetes, and hypertension, a one-unit increase in mother’s education was associated with an average increase of 0.021 T/S ratio in telomere length (regression coefficient *β* 0.021; 95% CI 0.001, 0.04, *p* =0.038). Regression coefficients for the confounders are provided in Supplementary Table 4. Once mediator variables (participant’s education level, physical activity, dietary habit, smoking status, depression score, and PSS) were introduced in the model, the estimate was reduced and remained marginally significant (*β* 0.017; 95% CI −0.003, 0.038, *p* = 0.061). Other individual components of the childhood SES, such as father’s education, parental occupation, parental homeownership, and family structure, had no association with TL.

**Table 3:** Associations between childhood SES indicators and leukocyte telomere length without (model 1) and with (model 2) additional adjustment for mediator variables, estimated from multivariable regression models (*n*=361): the GENE-FORECAST study

	Model1		Model 2	
	β (95% CI)	*P* value	β (95% CI)	*P* value
Mother’s education	0.021 (0.001, 0.04)	0.038	0.017 (−0.003, 0.038)	0.06
Father’s education	0.007 (−0.012, 0.026)	0.467	0.004 (−0.016, 0.023)	0.723
Mother’s occupation	−0.011 (−0.087, 0.066)	0.544	−0.027 (−0.105, 0.051),	0.493
Father’s occupation	−0.002 (−0.075, 0.077)	0.902	−0.003 (−0.08, 0.073)	0.926
Home ownership	0.011 (−0.061, 0.084)	0.759	0.004 (−0.071, 0.078)	0.923
Family structure	0.029 (−0.021, 0.081)	0.254	0.034 (−0.018, 0.087)	0.206

Values are multivariable-adjusted regression (*β*) coefficients (95% CIs)

Childhood SES indicators entered into the model as continuous variable

Model 1 adjusted for age, marital status, sex, BMI, and presence of hypertension and diabetes

Model 2 adjusted for the covariates of model 1 plus the specified mediator variables (education level, physical activity, dietary habit, smoking status, depression score, and PSS)

*SES* socioeconomic status, *PSS* Perceived Stress Score

**Table 4:** Estimation of indirect, direct, and total effects of childhood SES on telomere length and the proportion of the effects attributable to mediators from the path analysis: the GENE-FORECAST study

	^1^Direct effect		^2^Total indirect effect		^3^Total effect		^4^Percentage of mediation
	β (95% CI)	*P* value	β (95% CI)	*P* value	β (95% CI)	*P* value	
Mother’s education	0.017 (−0.003, 0.035)	0.06	0.004 (−0.001, 0.01)	0.10	0.021 (0.004, 0.036)	0.038	19.04%

Values are regression β coefficients (95% CIs) derived from path analysis. Mediation analyses were tested by using bootstrapping methods with bias-corrected confidence estimates

*SES* socioeconomic status, *TL* telomere length, *PSS* Perceived Stress Score

^1^Derivation of direct effects. In the path model, the effect size (coefficient) of a direct path is the regression coefficient between two variables

^2^Derivation of indirect effects. In the path model, the effect size (coefficient) of an indirect path is evaluated by calculating the product of the coefficients along that path

^3^Derivation of total effects. Direct and indirect effects were combined to derive the total effect

^4^The proportion of the associations between mother’s education and TL attributable to the mediator variables. Mediator variables are education level, physical activity, dietary habit, smoking status, depression score, and PSS of participants

Similar patterns of associations were noted when childhood SES was treated categorically (Supplementary Table 5). Compared to the reference group (lowest education group), participants whose mother had medium education (some college) had significantly longer TL both in model 1 (*β* 0.119; 95% CI 0.016, 0.222, *p* = 0.021) and in model 2 (*β* 0.118; 95% CI 0.015, 0.222, *p* = 0.025). Highest education group, however, showed marginal association with 0.071 T/S ratio longer TL compared the reference group in model 1 but did not reach statistical significance in model 2. We tested for the statistical interactions between participants’ age and mother’s education on participant’s TL. To examine the variation by gender, interaction between mother’s education and participant’s gender was also checked. To evaluate the interactions, we added main effects and cross-product terms to the regression after adjusting for all the confounding and mediator variables. *P* values for each interaction term and *F* tests comparing full and reduced models (with and without the interaction term) were used to test the statistical significance of the interaction terms. We found no evidence of effect modification by age or by gender in associations between mother’s education and TL.

### Path Analysis

**Fig. 1:**
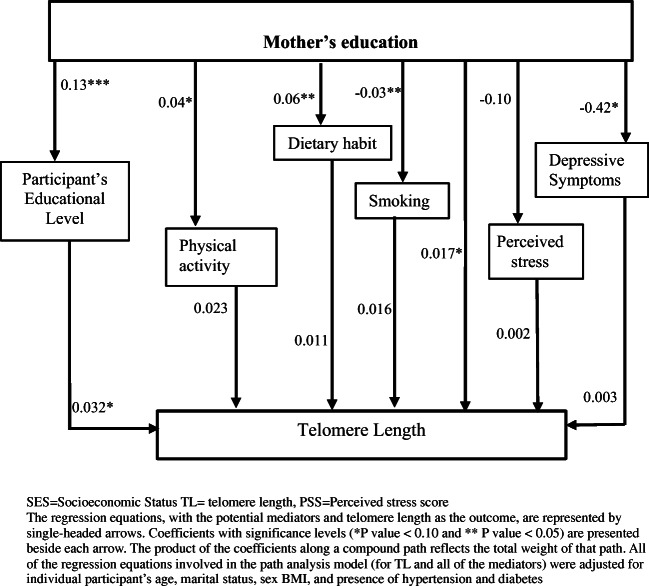
Estimated path analysis model showing the associations between mother’s education, potential mediators, and leukocyte TL (*n*=361): the GENE-FORECAST study

The path analysis model (Fig. 1) assessed the role played by potential mediators, explaining the associations between childhood SES indicators and adult TL. Path analyses were performed for mother’s education only, since the other SES indicators did not have any significant association with TL as seen in Table 3. The upper half of the path model shows the relation between mother’s education and the mediators and the lower half shows the effects of each individual mediator on the adult TL. According to the model, mother’s education was positively associated with mediators such as participant’s own education and their dietary habit and was negatively associated with participant’s smoking status. However, only one of these mediators, participant’s education (*β* 0.032; 95% CI −0.003, 0.068, *p* = 0.06), remained associated with TL at significance level of 0.10 as can be seen in the lower half of the path model, suggesting partial mediation by this factor. Therefore, the association between mother’s education and telomere length was not significantly mediated by diet, physical activity level, smoking habit, depression, or PSS in our sample. We also showed the estimated direct, indirect and total effects of mother’s education and composite score on adult TL with 95% CI from the path analysis in Table 4. As apparent from the path analysis, the associations between childhood SES and adult TL were mainly mediated by participant’s own education (About 19% mediation where total indirect effect: β: 0.004; 95% CI: -0.001, 0.01, *p* =0.09). RMSEA of the path models was 0.08 indicating a good fit with data. The NFI and the CFI estimates were however, lower than 0.90, indicating an acceptable model fit.

The result of sensitivity analysis (Supplementary Figure 3) shows that participant’s own education was associated with diet, smoking habit, depression, and PSS. However, none of these health behavior or psychological factors had any significant association with TL. Therefore, participant’s own education had no indirect or mediating effect through any of these factors.

### Social-Mobility Framework

For the social-mobility framework, we report mother’s educational level and compared trajectories with participant’s educational level as only mother’s education was significantly associated with adulthood TL. In our analysis, participants with a stable high SES (*β* 0.12; 95% CI 0.03,0.23, *p* =0.01) demonstrated having increased T/S ratio compared with women with a stable low SES (Table 5) after adjusting for all the confounders. After adjustment for all mediators as well, the association remained significant/strong (*β* 0.11; 95% CI 0.03, 0.26, *p* =0.02). For declining or increasing SES categories, no association was observed.

**Table 5:** Associations between social mobility of socioeconomic status leukocyte telomere length without (model 1) and with (model 2) additional adjustment for mediator variables, estimated from multivariable regression models (*n*=361): the GENE-FORECAST study

	Model1		Model 2	
Mother’s educational level/participant’s educational level	β (95% CI)	*P* value	β (95% CI)	*P* value
Low/low (stable low SES)	Reference		Reference	
High/low (declining SES)	0.016 (−0.081, 0.122)	0.624	0.006 (−0.081, 0.122)	0.738
Low/high (increasing SES)	0.039 (−0.057, 0.135)	0.427	0.033 (−0.081, 0.122)	0.288
High/high (stable high SES)	0.127 (0.029, 0.225)	0.008	0.121 (0.027, 0.241)	0.011

Values are multivariable-adjusted regression (*β*) coefficients (95% CIs)

Model 1 adjusted for age, marital status, sex, BMI, and presence of hypertension and diabetes

Model 2 adjusted for the covariates of model 1 plus the specified mediator variables (education level, physical activity, dietary habit, smoking status, depression score, and PSS)

*SES* socioeconomic status, *PSS* Perceived Stress Score

## Discussion

The present study provides evidence of a positive association between participant’s mother’s education and TL in AA adult population. Results from path analysis indicated that participant’s own educational level partially mediated this association but there was little evidence of mediation by participant’s health behavior and psychological factors. Other childhood SES measures had no association with TL. Social-mobility analyses indicated that participants with high mother’s education and high own education had longer TL compared to their lower SES counterparts.

Studies linking childhood SES to adult telomere length varied in the included age groups, study settings, and criteria for classifying childhood SES. Although few studies included AA participants, race-specific association was not available. Studies also used disparate sampling and analyzing methods to measure telomere length. Therefore, direct comparison of the existing literature with results of our study might not be always pertinent. Nevertheless, our findings are consistent with a recent study, where researchers found mother’s education to be associated with longer telomere length among a racially diverse sample of 81 pregnant women [36]. However, unlike ours, this study found association with father’s education as well. Parental homeownership had no impact on TL in our study participants. In contrast, shorter duration of parental homeownership was associated with adulthood shorter telomeres in a study of 135 White men and women [37]. Unlike ours, some studies reported effect modification by age. For example, two studies among Scottish and English White populations found that parental social class was linked with telomere length in a cohort of adults approximately age 35; however, this relationship was not observed among older cohorts, suggesting effect modifying role of participant’s age [32, 33].

Our results also suggested that the impact of mother’s education on adulthood TL was partially mediated by the participant’s own educational level. This finding was largely consistent with available literature supporting the pathway framework of early life socioeconomic circumstances on individuals’ SES and lifestyle in adulthood, which in turn impact adult health [38, 39]. With regard to the social-mobility SEP framework, we found participants with upward social mobility or with stable high SES (high mother’s and high own education) had longer TL than stable low SES (low mother’s and low own education). Prior studies have similarly reported that upward and stable high SES trajectories from childhood to adulthood were associated with decreased adulthood disease risk compared with a stable low SES trajectory [49, 50].

Several potential biological mechanisms may underlie the association we observed between mother’s education and participant’s TL. For example, exposure to socioeconomic burden in early life due to mother’s lower education may contribute to biological dysregulation of children through certain inflammatory regulators or through disruption of the hypothalamic-pituitary-adrenal axis causing epigenetic changes in glucocorticoid receptors [51]. This may interrupt the glucocorticoid activity, and increase inflammation and oxidative stress. Several studies have demonstrated that increased inflammations and oxidative stress are major risk factors of telomere shortening [9, 10]. It is also important to note that, cortisol, the most important glucocorticoid has been associated with telomere enzyme activity and can disrupt stabilizing TL [52]. Lastly, it is possible that genetic factors could also modulate the associations of mother’s education with adult TL [53]. TL is a highly heritable trait and is strongly influenced by genetic factors [54, 55]. It would be important for future studies to consider the role of genetic variants and genetic ancestry in modulating the associations of mother’s education with TL.

Although mother’s education was positively linked to adult TL in our analysis and the association was partially mediated through participant’s own education level, we did not find any mediating role of health behaviors, stress, and depression. Health behavior is a major determinant of morbidity and mortality in the USA and could strongly be patterned by childhood SES [56]. Associations of adult health behavior and psychological factors with TL have been examined previously, but the results have been inconsistent. For example, current smoking was associated with shorter TL in some [12, 13] but not all studies [14, 15]. Similarly, mixed results were reported for physical activity [14–16], dietary factors [14, 15, 17], and stress levels [22, 26, 27]. In our analysis, mother’s education was associated with most of these factors. But as none of them had any impact on TL after controlling for the confounding, they did not mediate the association between mother’s education and adult TL. The lack of association that we observed between the health behaviors, stress, and depression with TL could be due to the fact that majority of the variation in adult TL occurs very early in life, providing little opportunity for adult behavior to substantially impact TL [57]. It could also be due to cross-sectional nature of the study. Behavior patterns are associated with different rates of telomere attrition. We did not have longitudinal TL data to calculate the rate. We also did not have the measures of these health behavior over the life course to adjust for residual confounding. It is also possible that few of our variables, such as dietary habit and physical activity, where we used Single Global Questions did not capture the actual complexities of these variables and contributed to the diluting of the association.

We addressed both parent’s educational and professional attainment. This is important as most existing research focuses on father’s SES and often ignores mother’s SES. A meta-analysis of 29 studies examining the effects of low childhood SES on adult health found that father’s educational attainment and occupation was the most commonly used indicator of SES during childhood [58]. Although research has shown that both parent’s educational level can affect the health during childhood and adulthood, we found mother’s education to be associated with longer TL. The stronger association that we observed with mother’s education could be due to several underlying factors. During the past few decades, particular attention has been given to intrauterine conditions influencing the future health of the offspring. Unfavorable intrauterine conditions, which could be due to due to different maternal health-related factors like undernutrition, maternal stress, maternal smoking, and maternal exposure to toxins, not only affect immediate birth outcomes but also can lead to children’s aging-related disorders and telomere shortening [59, 60]. A number of research studies show that maternal health-related factors are directly caused by maternal unhealthy lifestyle and maternal education level. Mother’s education could also be a stronger determinant than their father’s education given the early development of behavior patterns in AA population. Studies showed that in AA families, mother’s involvement in parenting, and support are more stable in their distribution than father’s and thereby mother’s education could have more contribution on adult health of their children [61].

The main limitation of our study was its cross-sectional design and the lack of longitudinal data to assess whether childhood SES was related to the rate of change in telomere shortening or if there were any innate individual variation in telomere length. Therefore, longitudinal studies with repeated measures of TL are needed to determine whether the observed associations are causal and, if so, to identify the specific mechanisms involved. Also, our study measured telomere length only in leukocytes. Whether these associations can be extrapolated to all other tissues is unclear. Studies have, however, demonstrated strong correlations between TL and telomere length in other tissues, such as vascular wall, synovial tissue, skin, and umbilical artery [62, 63] Furthermore, TL measure using Cawthon’s qPCR technique is subject to measurement errors thus may mask true associations between mother’s education and TL, although this is less likely since our TL measure had higher accuracy with small coefficient of variations. The participants of the GENE FORECAST cohort are not representative of the general population of AAs with regard to the socio-demographic spectrum observed. In particular, people who experienced extreme poor SES in early life probably are underrepresented in this study. It has been suggested that adult recall of parental SES might be subject to error. Therefore, errors in remembering early life SES may also have biased results [64]. The continuous measures of SES relied on the assumption that ordinal or the interval-level of SES measurements were equally spaced. However, treating such variables, especially measures of education as continuous, is very common and well supported in literature [65].

The major strength of our study is that our data came from a well-characterized sample of African Americans, which is vastly understudied but has high prevalence of age-related chronic diseases including obesity, diabetes, hypertension, and others. To our knowledge, the current analysis is the first study to evaluate this association between childhood SES and adult TL explicitly and exclusively in AA participants; thus, our findings contribute new evidence to the limited knowledge in this research topic. We tested pathway and social mobility models and we found consistent results indicating that mother’s education impacts participant’s adult TL and participant’s own education plays a mediating or a joint effect in this association. Strength of the study also includes use of SES measures from six different aspects from both the parents which allowed for a more nuanced and comprehensive assessment. Most of the previous studies focused on a single childhood SES indicator from either maternal or paternal side. We used cross-sectional data for the present study. However, our study is strengthen by the fact that reverse causation between SES and adulthood TL is unlikely for the findings as childhood SES exposure preceded the adulthood TL.

In conclusion, the present study provides new evidence that childhood socioeconomic conditions, especially mother’s education, may have lasting consequences for aging and health throughout the life. The observations reported here are consistent with the hypothesis that telomere might link childhood SES to age-related chronic disease risk in AA population and speak to a need for further examining the role of other pathways with longitudinal data.

## Supplementary Information

ESM 1 (DOCX 162 kb)

## References

[1] Adler NE, Rehkopf DH (2008). U.S. disparities in health: descriptions, causes, and mechanisms. Annu Rev Public Health.

[2] Office of Minority Health Resource Center. “Profile: Black/African Americans.” Black/African American - The Office of Minority Health; 2019, www.minorityhealth.hhs.gov/omh/browse.aspx?lvl=3&lvlid=61.)

[3] Kittleson MM, Meoni LA, Wang N, Chu AY, Ford DE, Klag MJ (2006). Association of childhood socioeconomic status with subsequent coronary heart disease in physicians. Arch Intern Med.

[4] Braveman P, Barclay C (2009). Health disparities beginning in childhood: a life-course perspective. Pediatrics.

[5] Hornsby PJ (2007). Telomerase and the aging process. Exp Gerontol.

[6] Sahin E, DePinho RA (2012). Axis of ageing: telomeres, p53 and mitochondria. Nat Rev Mol Cell Biol.

[7] Blackburn EH (2000). Telomere states and cell fates. Nature.

[8] Blasco MA (2005). Telomeres and human disease: ageing, cancer and beyond. Nat Rev Genet.

[9] von Zglinicki T, Serra V, Lorenz M, Saretzki G, Lenzen-Groimlighaus R, Gener R (2000). Short telomeres in patients with vascular dementia: an indicator of low antioxidative capacity and a possible risk factor?. Lab Investig.

[10] von Zglinicki T (2002). Oxidative stress shortens telomeres. Trends Biochem Sci.

[11] Wolkowitz OM, Mellon SH, Epel ES, Lin J, Dhabhar FS, Su Y, Reus VI, Rosser R, Burke HM, Kupferman E, Compagnone M, Nelson JC, Blackburn EH (2011). Leukocyte telomere length in major depression: correlations with chronicity, inflammation and oxidative stress - preliminary findings. PLoS One.

[12] Khan RJ, Gebreab SY, Gaye A, Crespo PR, Xu R, Davis SK (2019). Associations of smoking indicators and cotinine levels with telomere length: National Health and Nutrition Examination Survey. Prev Med Rep.

[13] Huzen J, Wong LS, van Veldhuisen DJ, Samani NJ, Zwinderman AH, Codd V (2014). Telomere length loss due to smoking and metabolic traits. J Intern Med.

[14] Bekaert S, De Meyer T, Rietzschel ER, De Buyzere ML, De Bacquer D, Langlois M (2007). Telomere length and cardiovascular risk factors in a middle-aged population free of overt cardiovascular disease. Aging Cell.

[15] Cassidy A, De Vivo I, Liu Y, Han J, Prescott J, Hunter DJ (2010). Associations between diet, lifestyle factors, and telomere length in women. Am J Clin Nutr.

[16] Cherkas LF, Hunkin JL, Kato BS, Richards JB, Gardner JP, Surdulescu GL, Kimura M, Lu X, Spector TD, Aviv A (2008). The association between physical activity in leisure time and leukocyte telomere length. Arch Intern Med.

[17] Xu Q, Parks CG, DeRoo LA, Cawthon RM, Sandler DP, Chen H (2009). Multivitamin use and telomere length in women. Am J Clin Nutr.

[18] Epel ES, Blackburn EH, Lin J, Dhabhar FS, Adler NE, Morrow JD, Cawthon RM (2004). Accelerated telomere shortening in response to life stress. Proc Natl Acad Sci U S A.

[19] Zhang L, Hu XZ, Li X, Li H, Smerin S, Russell D, Ursano RJ (2014). Telomere length - a cellular aging marker for depression and Post-traumatic Stress Disorder. Med Hypotheses.

[20] Simon NM, Smoller JW, McNamara KL, Maser RS, Zalta AK, Pollack MH (2006). Telomere shortening and mood disorders: preliminary support for a chronic stress model of accelerated aging. Biol Psychiatry.

[21] Lung FW, Chen NC, Shu BC (2007). Genetic pathway of major depressive disorder in shortening telomeric length. Psychiatr Genet.

[22] Ridout KK, Ridout SJ, Price LH, Sen S, Tyrka AR (2016). Depression and telomere length: a meta-analysis. J Affect Disord.

[23] Sibille KT, Langaee T, Burkley B, Gong Y, Glover TL, King C (2012). Chronic pain, perceived stress, and cellular aging: an exploratory study. Mol Pain.

[24] Tomiyama AJ, O’Donovan A, Lin J, Puterman E, Lazaro A, Chan J (2012). Does cellular aging relate to patterns of allostasis? An examination of basal and stress reactive HPA axis activity and telomere length. Physiol Behav.

[25] Schutte NS, Malouff JM. The relationship between perceived stress and telomere length: a meta-analysis. Stress Health. 2014;13(10) 10.1002/smi.2607 25393133

[26] Needham BL, Mezuk B, Bareis N, Lin J, Blackburn EH, Epel ES (2015). Depression, anxiety and telomere length in young adults: evidence from the National Health and Nutrition Examination Survey. Mol Psychiatry.

[27] Mathur MB, Epel E, Kind S, Desai M, Parks CG, Sandler DP, Khazeni N (2016). Perceived stress and telomere length: a systematic review, meta-analysis, and methodologic considerations for advancing the field. Brain Behav Immun.

[28] Batty GD, Wang Y, Brouilette SW, Shiels P, Packard C, Moore J, Samani NJ, Ford I (2009). Socioeconomic status and telomere length: the West of Scotland Coronary Prevention Study. J Epidemiol Community Health.

[29] Needham BL, Adler N, Gregorich S, Rehkopf D, Lin J, Blackburn EH, Epel ES (2013). Socioeconomic status, health behavior, and leukocyte telomere length in the National Health and Nutrition Examination Survey, 1999-2002. Soc Sci Med.

[30] Cherkas LF, Aviv A, Valdes AM, Hunkin JL, Gardner JP, Surdulescu GL, Kimura M, Spector TD (2006). The effects of social status on biological aging as measured by white-blood-cell telomere length. Aging Cell.

[31] Carroll JE, Diez-Roux AV, Adler NE, Seeman TE (2013). Socioeconomic factors and leukocyte telomere length in a multi-ethnic sample: findings from the multi-ethnic study of atherosclerosis (MESA). Brain Behav Immun.

[32] Adams J, Martin-Ruiz C, Pearce MS, White M, Parker L, von Zglinicki T (2007). No association between socio-economic status and white blood cell telomere length. Aging Cell.

[33] Smith GD (2007). Life-course approaches to inequalities in adult chronic disease risk. Proc Nutr Soc.

[34] Beller E, Hout M (2006). Intergenerational social mobility: the United States in comparative perspective. Futur Child.

[35] Doom JR, Mason SM, Suglia SF, Clark CJ (2017). Pathways between childhood/adolescent adversity, adolescent socioeconomic status, and long-term cardiovascular disease risk in young adulthood. Soc Sci Med.

[36] Due P, Krølner R, Rasmussen M, Andersen A, Trab Damsgaard M, Graham H, Holstein BE (2011). Pathways and mechanisms in adolescence contribute to adult health inequalities. Scand J Public Health.

[37] Cohen S, Janicki-Deverts D, Turner RB, Marsland AL, Casselbrant ML, Li-Korotky H-S, Epel ES, Doyle WJ (2013). Childhood socioeconomic status, telomere length, and susceptibility to upper respiratory infection. Brain Behav Immun.

[38] Sternfeld B, Cauley J, Harlow S, Liu G, Lee M (2000). Assessment of physical activity with a single global question in a large, multiethnic sample of midlife women. Am J Epidemiol.

[39] Kuh D, Ben-Shlomo Y, Lynch J, Hallqvist J, Power C (2003). Life course epidemiology. J Epidemiol Community Health.

[40] Cohen S, Janicki-Deverts D, Chen E, Matthews KA (2010). Childhood socioeconomic status and adult health. Ann N Y Acad Sci.

[41] Diez Roux AV, Ranjit N, Jenny NS, Shea S, Cushman M, Fitzpatrick A, Seeman T (2009). Race/ethnicity and telomere length in the Multi-Ethnic Study of Atherosclerosis. Aging Cell.

[42] Hunt SC, Chen W, Gardner JP, Kimura M, Srinivasan SR, Eckfeldt JH, Berenson GS, Aviv A (2008). Leukocyte telomeres are longer in African Americans than in whites: the National Heart, Lung, and Blood Institute Family Heart Study and the Bogalusa Heart Study. Aging Cell.

[43] Rewak M, Buka S, Prescott J, De Vivo I, Loucks EB, Kawachi I, et al. Race-related health disparities and biological aging: does rate of telomere shortening differ across blacks and whites? Biol Psychol 2014;99:92-9. 10.1016/j.biopsycho.2014.03.007, 99. 10.1016/j.biopsycho.2014.03.007 PMC4610356 24686071

[44] Cawthon RM. Telomere measurement by quantitative PCR. Nucleic Acids Res. 2002, 30;(10):e47-e. 10.1093/nar/30.10.e47 PMC115301 12000852

[45] Callicott RJ, Womack JE (2006). Real-time PCR assay for measurement of mouse telomeres. Comp Med.

[46] Powell-Wiley TM, Miller PE, Agyemang P, Agurs-Collins T, Reedy J (2014). Perceived and objective diet quality in US adults: a cross-sectional analysis of the National Health and Nutrition Examination Survey (NHANES). Public Health Nutr.

[47] Radloff LS (1977). The CES-D scale a self-report depression scale for research in the general population. Appl Psychol Meas.

[48] Cohen S, Kessler RC, Underwood Gordon L (1995). Perceived stress scale. Measuring stress: a guide for health and social scientists.

[49] Lidfeldt J, Li TY, Hu FB, Manson JE, Kawachi I (2007). A prospective study of childhood and adult socioeconomic status and incidence of type 2 diabetes in women. Am J Epidemiol.

[50] Regidor E, Banegas JR, Gutiérrez-Fisac JL, Domínguez V, Rodríguez-Artalejo F (2004). Socioeconomic position in childhood and cardiovascular risk factors in older Spanish people. Int J Epidemiol.

[51] Morris G, Berk M, Maes M, Carvalho AF, Puri BK (2019). Socioeconomic deprivation, adverse childhood experiences and medical disorders in adulthood: mechanisms and associations. Mol Neurobiol.

[52] Valdes AM, Andrew T, Gardner JP, Kimura M, Oelsner E, Cherkas LF, Aviv A, Spector TD (2005). Obesity, cigarette smoking, and telomere length in women. Lancet.

[53] Mitchell C, Hobcraft J, McLanahan SS, Siegel SR, Berg A, Brooks-Gunn J, et al. Social disadvantage, genetic sensitivity, and children’s telomere length. Proc Natl Acad Sci. 2014;(111, 16):5944–9. 10.1073/pnas.1404293111. 10.1073/pnas.1404293111 PMC4000782 24711381

[54] Andrew T, Aviv A, Falchi M, Surdulescu GL, Gardner JP, Lu X, Kimura M, Kato BS, Valdes AM, Spector TD (2006). Mapping genetic loci that determine leukocyte telomere length in a large sample of unselected female sibling pairs. Am J Hum Genet.

[55] Honig LS, Kang MS, Cheng R, Eckfeldt JH, Thyagarajan B, Leiendecker-Foster C, Province MA, Sanders JL, Perls T, Christensen K, Lee JH, Mayeux R, Schupf N (2015). Heritability of telomere length in a study of long-lived families. Neurobiol Aging.

[56] Ford ES, Zhao G, Tsai J, Li C (2011). Low-risk lifestyle behaviors and all-cause mortality: findings from the National Health and Nutrition Examination Survey III Mortality Study. Am J Public Health.

[57] Benetos A, Kark JD, Susser E, Kimura M, Sinnreich R, Chen W, Steenstrup T, Christensen K, Herbig U, von Bornemann Hjelmborg J, Srinivasan SR, Berenson GS, Labat C, Aviv A (2013). Tracking and fixed ranking of leukocyte telomere length across the adult life course. Aging Cell.

[58] Galobardes B, Lynch JW, Davey SG (2004). Childhood socioeconomic circumstances and cause-specific mortality in adulthood: systematic review and interpretation. Epidemiol Rev.

[59] Barker DJ (1998). In utero programming of chronic disease. Clin Sci.

[60] Shankar K, Harrell A, Liu X, Gilchrist JM, Ronis MJ, Badger TM (2008). Maternal obesity at conception programs obesity in the offspring. Am J Physiol Regul Integr Comp Physiol.

[61] Bean RA, Barber BK, Crane DR (2006). Parental support, behavioral control, and psychological control among African American youth: the relationships to academic grades, delinquency, and depression. J Fam Issues.

[62] Okuda K, Bardeguez A, Gardner JP, Rodriguez P, Ganesh V, Kimura M, Skurnick J, Awad G, Aviv A (2002). Telomere length in the newborn. Pediatr Res.

[63] Wilson WRW, Herbert KE, Mistry Y, Stevens SE, Patel HR, Hastings RA, Thompson MM, Williams B (2008). Blood leucocyte telomere DNA content predicts vascular telomere DNA content in humans with and without vascular disease. Eur Heart J.

[64] Batty GD, Lawlor DA, Macintyre S, Clark H, Leon DA (2005). Accuracy of adults’ recall of childhood social class: findings from the Aberdeen children of the 1950s study. J Epidemiol Community Health.

[65] Jordá V, Alonso JJaA. Measuring educational attainment as a continuous variable: a new database (1970-2010). 2015

