# Supplementary material for: Association of Childhood Socioeconomic Status with Leukocyte Telomere Length Among African Americans and the Mediating Role of Behavioral and Psychosocial Factors: Results from the GENE-FORECAST Study
Source: J Racial Ethn Health Disparities. 2021 May 4;9(3):1012–23. doi: 10.1007/s40615-021-01040-5 (PMC9061663; doi:10.1007/s40615-021-01040-5)
Supplement: Supplementary file 1 — (DOCX 162 kb) [file 40615_2021_1040_MOESM1_ESM.docx]

**Title: Association of childhood socioeconomic status with leukocyte telomere length among African Americans and the mediating role of behavioral and psychosocial factors: Results from the GENE-FORECAST study**

**Corresponding author**

Rumana J Khan

Cardiovascular Section

Social and Behavioral Research Branch, National Human Genome Research Institute, National Institutes of Health, 10 Center Drive, Room 7N316 MSC 1644, Bethesda, MD 20892

Phone: +1301-827-8350

Email: rumana.khan@nih.gov, rjkhan@ucdavis.edu

**Author names**

Rumana J Khan^1^

Belinda Needham^2^

Kristen Brown^1^

Casey Dagnall^3^

Ruihua Xu^1^

Gary H Gibbons^4,5^

Sharon K Davis^1^

**Supplementary Table1** Scoring of childhood socioeconomic status

|  | **Question** | **Scoring** |
| --- | --- | --- |
| **Mother’s education** | What is the highest degree or years of school your mother (or important female caretaker) completed, including trade or vocational school or college? | 0, 8th grade or below  1, 9th to 12th grades,  2, high school diploma/ equivalent,  3, Vocational/Tech program  4, some college,  5, bachelor’s degree,  6, Master’s degree  7, Doctorate degree |
| **Mother’s occupation** | When you were growing up, until you were 16 years old or so, what was your mother’s (or other important mother caretaker’s) main job (the most important one)? | 0, mother was involved in nonprofessional work,  1, if she was either doing professional or managerial work |
| **Father’s education** | What is the highest degree or years of school your father (or important male caretaker) completed, including trade or vocational school or college? | 0, 8th grade or below  1, 9th to 12th grades,  2, high school diploma/ equivalent,  3, Vocational/Tech program  4, some college,  5, bachelor’s degree,  6, Master’s degree  7, Doctorate degree |
| **Father’s occupation** | When you were growing up, until you were 16 year old or so, what was your father’s (or other important male caretaker’s) main job (the most important one)? | 0, mother was involved in nonprofessional work,  1, if she was either doing professional or managerial work |
| **Parent Housing** | When you were growing up, until you were 16 years old or so, did your parents (or people who raised you) own or were buying their home, pay rent, or had some other living arrangement, such as living with relatives, etc,? | 0, lived with relatives/others  1, had rented house  2, had own house |
| **Caregiver** | Were you raised up to age 16 by Both parents/ Single mother/Single father/Female caregiver other than your mother/Male caretaker other than your father | 0 raised by someone other than parents.  1 raised by single father  2, raised by single mother  3, raised by both parents |
| **Cumulative childhood SES** | A summary score of overall childhood SES created by summing the points of above six measures | 0-21 |

*For sensitivity analysis, parental educational level was categorized as i) 0-12 years/ high school diploma (score 1 to 3, low education), ii) Some college (score 4 and 5, medium education), and iii) 4 year college or higher (score 6 to 8, high education). Parental occupation as non-professional/homemaker vs managerial/professional. Parental house was categorized as lived with relatives or others (reference), had rented house, or had owned house, and family structure was categorized as raised by someone other than parents (reference), raised by single father, raised by single mother, or raised by both parents.*

**Relative telomere length (RTL) measurement**

Relative telomere length (RTL) measurement was performed on DNA isolated from peripheral blood leukocytes at the Cancer Genomics Research (CGR) laboratory, National Cancer Institute, using an assay adapted from Cawthon’s published protocol (1). Relative telomere length determination by qPCR measures the ratio of telomere (T) signals, specific to the telomere hexamer repeat sequence TTAGGG, to autosomal single copy gene (S) signals. This ratio is normalized by control DNA samples to yield relative standardized T/S ratios proportional to average telomere length. In this technique, reactions are performed independently, so a standard curve of pooled DNA samples is utilized to assess the amount of each signal, while compensating for inter-late variations in PCR efficiency.

For this analysis, in each reaction, 4 ng of sample DNA, according to Quant-iT PicoGreen dsDNA quantiation (Life Technologies, Grand Island, NY), was transferred into LightCycler-compatible 384-well plates (Roche, Indianapolis, IN) and dried down. A standard curve [6 concentrations of pooled reference DNA samples prepared by serial dilution (4 to 0.4096 ng/uL) was added in triplicate to each 384-well plate. Randomly located internal QC sample replicates (n=5), utilized as calibrator samples, to guide analysis and indicate overall quality of assay performance were added to each sample plate. Additionally, an NTC was added to a random location on each sample plate to provide a unique fingerprint for each plate. All experimental and control samples were assayed in triplicate on each 384-well assay plate for both assays. All pipetting steps were performed using a Biomek FX (Beckman Coulter, Indianapolis, IN) liquid handler calibrated to perform transfers from 2-50 uL with a CV of <5%.

Primers for the telomeric PCR were Telo_FP [5′-CGGTTT(GTTTGG)5GTT-3′] and Telo_RP [5′-GGCTTG(CCTTAC)5CCT-3′] [2]. Primers for the single-copy gene (36B4) PCR were 36B4_FP [5′-CAGCAAGTGGGAAGGTGTAATCC-3′] and 36B4_RP [5′ CCCATTCTATCATCAACGGGTACAA-3′] [1]. Primers were manufactured LabReady, normalized to 100 uM in IDTE, pH 8.0 and HPLC Purified (Integrated DNA Technologies, Coralville, IA). 1 uM assay mixes for each target were generated by combining 990 uL of 1X Tris-EDTA Buffer with 5 uL of forward oligo and 5 uL of reverse oligo.

PCR was performed using 5 uL reaction volumes consisting of: 2.5 uL of 2X Rotor-Gene SYBR Green PCR Master Mix (Qiagen, Germantown, MD), 2.0 uL of molecular biology grade water, and .5 uL of 1 µM assay-specific mix of primers. Thermal cycling was performed on a LightCycler 480 (Roche) where PCR conditions were (i) T (telomeric) PCR: 95°C hold for 5 min, followed by 35 cycles of denature at 98°C for 15 sec and anneal at 54°C for 2 min, with fluorescence data collection and (ii) S (single-copy gene, 36B4) PCR: 98°C hold for 5 min, followed by 43 cycles of denature at 98°C for 15 sec and anneal at 58°C for 1 min, with fluorescence data collection.

The LightCycler software (Release 1.5.0, Roche, Indianapolis, IN, USA) was used to generate Ct values, utilizing absolute quantification analysis with the second derivative maximum method and high sensitivity detection algorithm. Ct values of triplicates were averaged, if meeting a CV threshold of less than 2%, and the concentration (ng/uL) was interpolated from the plate-specific standard curve’s exponential regression [Average Ct and log2(Concentration)]. Samples with 36B4 concentrations falling outside the range of the standard curve were dropped from further analysis. In this dataset the mean CV of triplicates for the telomere assay was 0.510753% and for the 36B4 assay was 0.30992%. The telomere (T) concentration was divided by the 36B4 (S) concentration to yield a raw T/S ratio. The raw T/S ratio is divided by the average raw T/S ratio of the internal QC calibrator samples, within the same plate, to yield a standardized T/S ratio to normalize results in reference to the same individual. The mean coefficient of variations (CV) for the standardized T/S measure of technical replicates from 58 subjects was 6.09801% across all projects (timepoints) and tests.

Within this dataset there were also technical replicates of GENE-FORECAST samples, resulting in multiples instances or results within and/or between projects (timepoints). In total, for this dataset, there were, 32 samples which have 2 data points, 20 samples which have 5 data points, 3 samples which have 6 data points, 2 samples which have 22 data points and the average was used for final calculations. Additionally, the standardized T/S ratio was calculated using two GENE-FORECAST samples that were repeated 22 times as internal calibrator samples (internal replicate), which provided a more accurate assessment of RTL given the dynamic range GENE-FORECAST samples

A brief statistic of all the 3 standardization procedures of raw T/S ratio is summarized below:

|  | **Coefficient of variation (CV %)** | **intraclass correlation coefficient (ICC, 95% CI)** |
| --- | --- | --- |
| NA07057 (Cancer Genome Research Internal Control) | 6.09% | 0.920, 0.898, 0.936 |
| GENE-FORECAST internal control sample 1 | 5.36% | 0.909, 0.885, 0.928 |
| GENE-FORECAST internal control sample 2 | 4.90% | 0.902, 0.876, 0.922 |

**References**

1. Cawthon RM. Telomere measurement by quantitative PCR. Nucleic Acids Research. 2002;30(10):e47-e.

2. Callicott, RJ and JE Womack. Real-time PCR assay for measurement of mouse telomeres. Computational Medicine. 2006;56,17-22.

**Measurement of the covariates:**

Participant’s educational attainment was scored, ranging from 1 to 8 with higher values corresponding to higher education. Smoking data was self-reported. Participants were categorized as never, former and current smokers. Participants were asked as to what they thought about their physical activity during leisure time in comparison with others of their own age. The scale ranged from 1-5, where higher values indicated greater physical activity. Dietary score was also assigned as scales that ranged from 1 (if they self-rated their current diet as poor) to 5 (if they rated their current diet as excellent). The 20‐item Center for Epidemiologic Studies Depression Scale (CES‐D) was used to assess the presence of depressive symptoms. The score ranged from 0-44, with higher score indicating more depressive symptoms. Perceived Stress was assessed using the 10-item self-reported Perceived Stress Scale (PSS) questionnaire, which assesses individual’s perceptions of psychological stress during the past month and the score ranged from 0-34. Higher scores corresponded to higher stress level.

**References:**

Sternfeld B, Cauley J, Harlow S, Liu G, Lee M. Assessment of Physical Activity with a Single Global Question in a Large, Multiethnic Sample of Midlife Women. American Journal of Epidemiology. 2000;152(7):678-87.

Powell-Wiley TM, Miller PE, Agyemang P, Agurs-Collins T, Reedy J. Perceived and objective diet quality in US adults: a cross-sectional analysis of the National Health and Nutrition Examination Survey (NHANES). Public Health Nutrition. 2014;17(12):2641-9.

Radloff LS. The CES-D scale a self-report depression scale for research in the general population. Applied psychological measurement. 1977;1(3):385-401.

Cohen S, Kessler RC, Underwood Gordon L: Perceived stress scale. Measuring Stress: A Guide for Health and Social Scientists. 1995, New York: Oxford University Press.

**Supplementary Table 2** Measurement of the covariates:

| **Confounder** |  |
| --- | --- |
| Age | In years |
| Sex | Male/female |
| Marital status | Partnered/ non partnered |
| Disease condition (hypertension, BMI, diabetes) | Yes/no for hypertension and diabetes |
| **Mediators** |  |
| ^1^participant’s education | 1, no school/only kindergarten,  2, grades 1 through 8 (elementary),  3 grades 9 through 11 (some high school),  4, grade 12 or GED (high school graduate),  5, college 1 year to 3 years (some college or technical school),  6, college 4 years or more (college graduate),  7, Master’s Degree and  8, Doctoral Degree (PhD, EdD, MD, JD, ScD). |
| smoking status | 1=never, 2= former, 3=current |
| physical activity | 1, Much less than others  2, Less than others  3, The same as others  4, More than others  5, Much more than others |
| dietary habit | 1, Poor  2, Fair  3, Good  4, Very good  5, Excellent |
|  |  |
| perceived stress score | 10-item self-reported Perceived Stress Scale (PSS) questionnaire, which assess individual’s perception of psychological stress during the past month |
| depressive symptom score | The 20‐item Center for Epidemiologic Studies Depression Scale (CES‐D) to assess the presence of depressive symptoms |

*^1^For descriptive statistics educational level was categorized as i) 0-12 years/ high school diploma (score 1 to 4), ii) Some college (score 4 and 5), and iii) 4 year college or higher (score 6 to 8)*

*Missing: Own education 2, Smoking status 5, Body mass index 15, Physical activity 4, Diet 3, Depressive symptoms 2*

**Path analysis model:**

Supplementary Figure 1 a shows the proposed path analysis model through which childhood SES may directly or indirectly impact adult telomere length. To investigate the extent to which the intervening variables mediate the association between childhood SES measures and telomere length, we performed path analysis by using Analysis of Moment Structures (AMOS). Path analysis simultaneously estimated the regression of the outcome (telomere length) on the respective mediators and the regression of each of those mediators on exposure (childhood SES measures). This method thus allowed us to decompose the total effect of childhood SES on telomere length into a direct effect and an indirect effect that may act via intervening variables as shown in Figure 1. All of the regression equations involved in the path analysis model were adjusted for participant’s age, sex, marital status, and presence of disease conditions including obesity, hypertension and diabetes. Mediation effects was estimated by using bootstrapping methods (sample = 1000), and bias-corrected confidence interval (CI) estimates for the indirect effects were obtained. The comparative fit index, normal fit index, and the root mean square error of approximation (RMSEA) were used to evaluate the model fit of the path analysis. All the variables of the model were checked for significant associations with each other, and a correlation matrix was derived by using all of the variables in the model (Supplementary Table 3).

**Supplementary Figute1** Path analysis model showing the associations between Childhood SES, potential mediators, and telomere length: the GENE-FORECAST Study

childhood SES

Dietary habit

Participant’s Educational Level

Depressive Symptoms

Perceived stress

Smoking

Physical

activity

Telomere Length

**Supplementary Figure 2** Path analysis model showing the associations between Childhood SES, potential mediators, and telomere length: the GENE-FORECAST Study (Sensitivity analysis)

**Mother’s education**

Dietary habit

Participant’s Educational Level

Depressive Symptoms

Smoking

Perceived stress

Physical

activity

Telomere Length

**Supplementary Table 3** Correlation coefficients among indicators of childhood SES, proposed mediator variables and leukocyte TL^1^

|  | Mother’ education | Father’s education | Parental Home ownership | Family Structure | Cumulative Childhood SES |
| --- | --- | --- | --- | --- | --- |
| Participant’s education | 0.213*** | .225*** | .165*** | -.047 | .241*** |
| Physical activity | 0.06* | .029 | .034 | -.087 | 0.076 |
| Diet | 0.05 | .054 | .155*** | -.137*** | 0.092 |
| Smoking | -0.122** | -0.156*** | -.098 | -0.156*** | -0.171*** |
| Depression | -0.043 | -.073 | -0.149*** | -0.121** | -0.104** |
| PSS | 0.02 | -.015 | -0.053 | -0.137** | -0.029 |
| Telomere | 0.178*** | 0.104** | 0.006 | -0.02 | 0.134** |

*^1^Coefficients with significance levels *P value < 0.10, ** P value < 0.05 and *** P value < 0.01.*

*SES=Socioeconomic Status, TL= Telomere length, PSS=Perceived stress score*

**Supplementary Table 4** Associations between Childhood SES and leukocyte telomere length without (model 1) and with (model 2) additional adjustment for mediator variables, estimated from multivariable regression models (n=361): the GENE-FORECAST study

|  | **Model1** | | **Model 2** | |
| --- | --- | --- | --- | --- |
|  | **Β (95% CI)** | **P value** | **Β (95% CI)** | **P value** |
| **Mother’s education** | 0.021(0.001, 0.04) | 0.038 | 0.017 (-0.003, 0.038) | 0.06 |
| **Confounding variables on TL** |  |  |  |  |
| Age | -0.006 (-0.010,-0.003) | 0.001 | 0.005 (-0.011, -0.003) | 0.001 |
| Not married | -.027(-0.108, 0.053) | 0.507 | -0.035 (-0.117, 0.047) | 0.400 |
| Female | 0.108 (0.028, 0.188) | 0.008 | 0.098 (0.016, 0.179) | 0.019 |
| BMI | -0.002 (-0.002, 0.001) | 0.515 | 0.001(-0.002, 0.001) | 0.693 |
| Hypertensive | -0.058 (-0.136, 0.021) | 0.150 | -0.052 (-0.131, 0.027) | 0.198 |
| Diabetic | 0.038 (-0.092, 0.168) | 0.566 | 0.040 (-0.091, 0.171) | 0.549 |

*SES=Socioeconomic Status, PSS=Perceived stress score, TL= telomere length, BMI=Body mass index*

*Values are multivariable-adjusted regression (b) coefficients (95% CIs),*

*Model 1 adjusted for age, marital status, sex, BMI, and presence of hypertension and diabetes*

*Model 2 adjusted for the covariates of model 1 plus the specified mediator variables (education level, physical activity, dietary habit, smoking status, depression score and PSS*

**Supplementary Table 5** Associations between childhood SES indicators and telomere length without (model 1) and with (model 2) additional adjustment for mediator variables, estimated from multivariable regression models (n=361): the GENE-FORECAST study

|  | **Model 1**  **β (95% CI), p** | **Model 2**  **β (95% CI), p** |
| --- | --- | --- |
| **Father’s Education** |  |  |
| Low | reference | reference |
| Medium | 0.038 (-0.07, 0.146), 0.489 | 0.013 (-0.096, 0.123), 0.81 |
| High | 0.02 (-0.068, 0.109), 0.652 | 0.005 (-0.086, 0.097), 0.91 |
| **Mother’s Education** |  |  |
| Low | reference |  |
| Medium | 0.119 (0.016, 0.222), 0.023 | 0.118 (0.015, 0.222), 0.025 |
| High | 0.071 (-0.015, 0.152), 0.08 | 0.053 (-0.036, 0.144), 0.244 |
| **Home ownership** |  |  |
| Lived with relatives/other | reference | reference |
| Lived in a rented house | 0.165 (-0.148, 0.490), 0.302 | 0.142 (-0.173, 0.458), 0.376 |
| Lived in own house | 0.161 (-0.150, 0.473), 0.310 | 0.131 (-0.181, 0.443), 0.410 |
| **Family structure/ raised by** |  |  |
| Someone other than parents | reference | reference |
| Single father | 0.242 (-0.034, 0.52), 0.09 | 0.215 (-0.062, 0.493), 0.128 |
| Single mother | 0.110 (-0.110, 0.331), 0.326 | 0.09 (-0.131, 0.312), 0.423 |
| Both Parents | 0.162(-0.049, 0.375), 0.133 | 0.155 (-0.059, 0.369), 0.155 |

*SES=Socioeconomic Status, PSS=Perceived stress score*

*Values are multivariable-adjusted regression (β) coefficients (95% CIs), p*

*Childhood SES indicators entered into the model as categorical variable*

*Parental educational level was categorized as low (0-12 years/ high school diploma), medium (some college) and high (4 year college or higher); and their occupation as non-professional/homemaker vs managerial/professional. Parental house was categorized as lived with relatives or others (reference), had rented house, or had owned house, and family structure was categorized as raised by someone other than parents (reference), raised by single father, raised by single mother, or raised by both parents.*

*Model 1 adjusted for age, sex, obesity, and presence of hypertension and diabetes*

*Model 2 adjusted for the covariates of model 1 plus the specified mediator variables (education level, physical activity, dietary habit, smoking status, depression score and PSS*

**Supplementary Figure 3** Estimated path analysis model showing the associations between mother’s education, potential mediators, and LTL (n=361): the GENE-FORECAST study

**Mother’s education**

0.13***

-0.10

-0.03**

-0.26

0.05**

0.04*

0.12**

Dietary habit

Participant’s Educational Level

Depressive Symptoms

Smoking

-0.14**

Perceived stress

0.017*

0.013

Physical

activity

0.002

0.023

0.016

-0.73**

-1.12**

0.011

0.003

0.033*

Telomere Length

*SES=Socioeconomic Status LTL= leukocyte telomere length, PSS=Perceived stress score*

*The regression equations, with the potential mediators and telomere length as the outcome, are represented by single-headed arrows. Coefficients with significance levels (*P value < 0.10 and ** P value < 0.05) are presented beside each arrow. The product of the coefficients along a compound path reflects the total weight of that path. All of the regression equations involved in the path analysis model (for LTL and all of the mediators) were adjusted for individual participant’s age, marital status, sex, BMI, and presence of hypertension and diabetes*
